# Case Report: Massive Hepatocellular Carcinoma Complete Surgical Resection After Portal Vein Embolization and Multimodality Therapy

**DOI:** 10.3389/fradi.2022.858963

**Published:** 2022-04-29

**Authors:** Qianyi Lin, Dexiong Chen, Kangde Li, Xiaomin Fan, Qi Cai, Weihong Lin, Chunhong Qin, Tao He

**Affiliations:** ^1^Department of Liver Surgery, Zhanjiang Central Hospital, Guangdong Medical University, Zhanjiang, China; ^2^Department of Pathology, Zhanjiang Central Hospital, Guangdong Medical University, Zhanjiang, China

**Keywords:** hepatocellular carcinoma, portal vein embolization (PVE), hepatic artery infusion chemotherapy, tyrosine kinase inhibitor (TKI), programmed cell death-1 inhibitor, case report

## Abstract

A high proportion of massive patients with hepatocellular carcinoma (HCC) are not amenable for surgical resection at initial diagnosis, owing to insufficient future liver remnant (FLR) or an inadequate surgical margin. For such patients, portal vein embolization (PVE) is an essential approach to allow liver hypertrophy and prepare for subsequent surgery. However, the conversion resection rate of PVE only is unsatisfactory because of tumor progression while awaiting liver hypertrophy. We report here a successfully treated case of primary massive HCC, where surgical resection was completed after PVE and multimodality therapy, comprising hepatic artery infusion chemotherapy (HAIC), Lenvatinib plus Sintilimab. A pathologic complete response was achieved. This case demonstrates for the first time that combined PVE with multimodality therapy appears to be safe and effective for massive, potentially resectable HCC and can produce deep pathological remission in a primary tumor.

## Introduction

Patients with hepatocellular carcinoma (HCC) with a solitary massive (≥10 cm) tumor, beyond Milan criteria, are not eligible for ablation or liver transplantation (1). Major hepatectomy remains the only potential radical treatment for such patients (1). However, insufficient future liver remnant (FLR) or an inadequate surgical margin hinders the implementation of major hepatectomy, especially for fibrotic livers (2). Insufficient FLR could result in post-hepatectomy liver failure (PHLF), which is a main cause of perioperative mortality. Safe hepatectomy requires a minimal FLR of 25–30% in the normal liver, compared with at least 40% in the cirrhotic liver (3). Moreover, a narrow surgical margin is an unfavorable prognostic factor (4).

Portal vein embolization (PVE) and associated liver partition and portal vein ligation for staged hepatectomy (ALPPS) have been proved to be effective in inducing FLR hypertrophy in a timely manner (2). Compared to ALPPS, PVE shows a lower risk of mortality and morbidity at the cost of a longer duration of liver hypertrophy, during which tumor progression may occur in ~20–40% of patients (5–7). Therefore, concurrent anticancer therapy is required as PVE proceeds. As a promising conversion therapy regimen, hepatic artery infusion chemotherapy (HAIC) has been proved to be safe and effective in patients with massive unresectable HCC with a resultantly higher conversion surgery rate than transarterial chemoembolization (TACE) (8). In recent years, a combination of tyrosine kinase inhibitors (TKI) and programmed cell death protein-1 (PD-1) inhibitors has been explored as conversion therapy for unresectable HCCs, producing obvious tumor shrinkage and an uplift to survival (9). These findings imply that PVE in combination with HAIC and TKI plus PD-1 inhibitors may have further conversion potential in treating potentially resectable HCCs with insufficient FLR or an inadequate surgical margin.

To date, rare cases have reported the efficacy and safety of combined PVE with multimodality therapy in potentially resectable HCC. We report here a successfully treated patient with primary massive HCC who underwent laparoscopic right hemi-hepatectomy after PVE and multimodality therapy and achieved a pathologic complete response (pCR).

## Case Presentation

**Figure 1:**
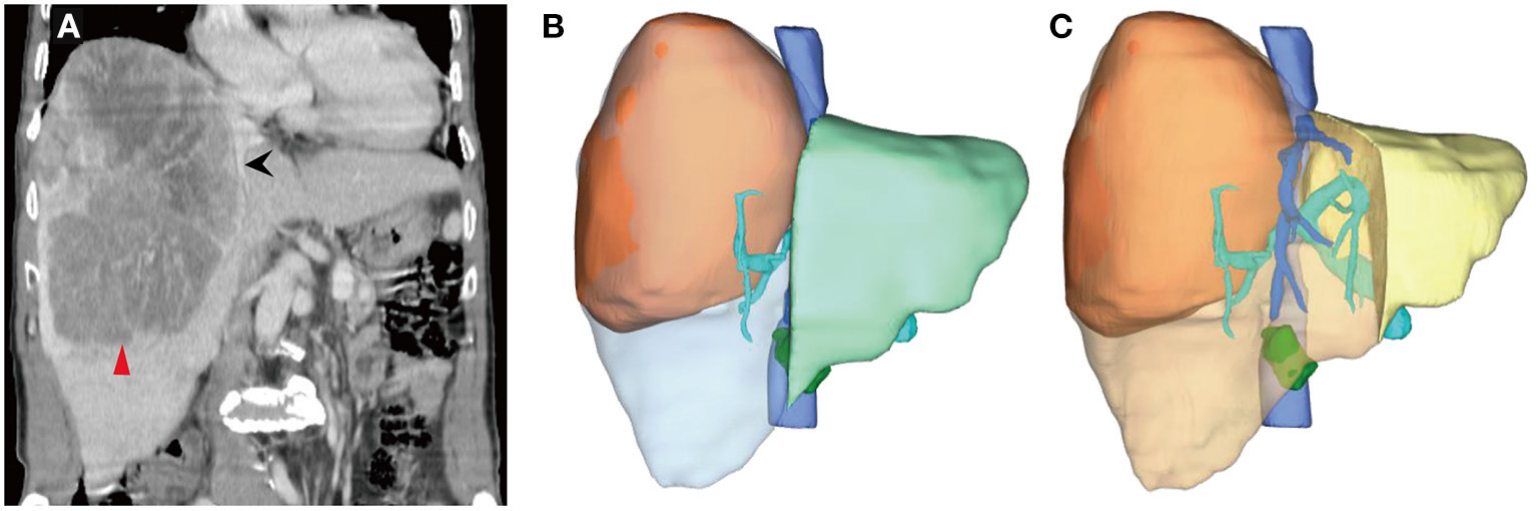
Computed tomography (CT) scans and three-dimensional reconstruction at initial diagnosis. **(A)** A large lesion (the red arrow head) in the right lobe of the liver, measuring about 19.3 × 14.6 × 13.2 cm. The middle hepatic vein (the black arrow) ran almost along the edge of the lesion. **(B)** The future liver remnant (FLR, the light green area), followed right hemi-hepatectomy divided by standard liver volume (SLV), was 51.47%, which was well-tolerated for hepatectomy, although the surgical margin was nearly zero. **(C)** If extended right hemi-hepatectomy were to be performed, the FLR (the yellow area)/SLV would be 25.78%, with an extremely high risk of post-hepatectomy liver failure.

A 74-year-old man presented to our hospital with nausea and vomiting for 2 weeks, without any history of gastrointestinal disorders. He had a history of alcoholism for over 20 years. An enlarged liver was palpable from the right hypochondrium without obvious jaundice or anemia. Contrast-enhanced computed tomography (CT) scans revealed a mass located in the right lobe of the liver (Figure 1A), measuring approximately 19.3 × 14.6 × 13.2 cm, with enhancement in the arterial phase and rapid washout in the portal phase, suggesting a diagnosis of primary liver cancer. No evidence of significant macrovascular invasion or intra- or extrahepatic metastases was observed on the CT scan. The patient was negative for both hepatitis B surface antigen and hepatitis C virus antibody. Des-gamma-carboxy prothrombin (DCP) was markedly elevated, whereas α-fetoprotein (AFP), carcinoembryonic antigen (CEA), and carbohydrate antigen 199 (CA 199) were normal. The Child-Pugh score was 5. To obtain definite histological evidence of the tumor and assess the extent of liver cirrhosis, a biopsy was performed. Finally, the patient was diagnosed as HCC with mild liver cirrhosis and staged as BCLC-A in the Barcelona Clinic Liver Cancer (BCLC) staging system. The etiology of HCC was chronic alcoholic hepatitis.

The ratio of FLR to standard liver volume (SLV) after anatomical right hemi-hepatectomy was 51.47% (Figure 1B), which was well-tolerated for hepatectomy (>40%). However, the middle hepatic vein ran almost along the edge of the lesion (Figure 1A). If a right hemi-hepatectomy were to be performed, it would be hard to ensure an adequate surgical margin. A narrow surgical margin has been proved to be an unfavorable prognostic factor. By contrast, an extended right hemi-hepatectomy would probably result in insufficient FLR (Figure 1C, FLR/SLV = 25.78%, far <40%), with a high risk of PHLF. Nevertheless, the patient wished to undergo surgical treatment. After discussion among the multidisciplinary team (MDT), a combination of PVE and multimodality therapies, comprising HAIC, Lenvatinib, and Sintilimab, was considered the optimal choice in the circumstances.

**Figure 2:**
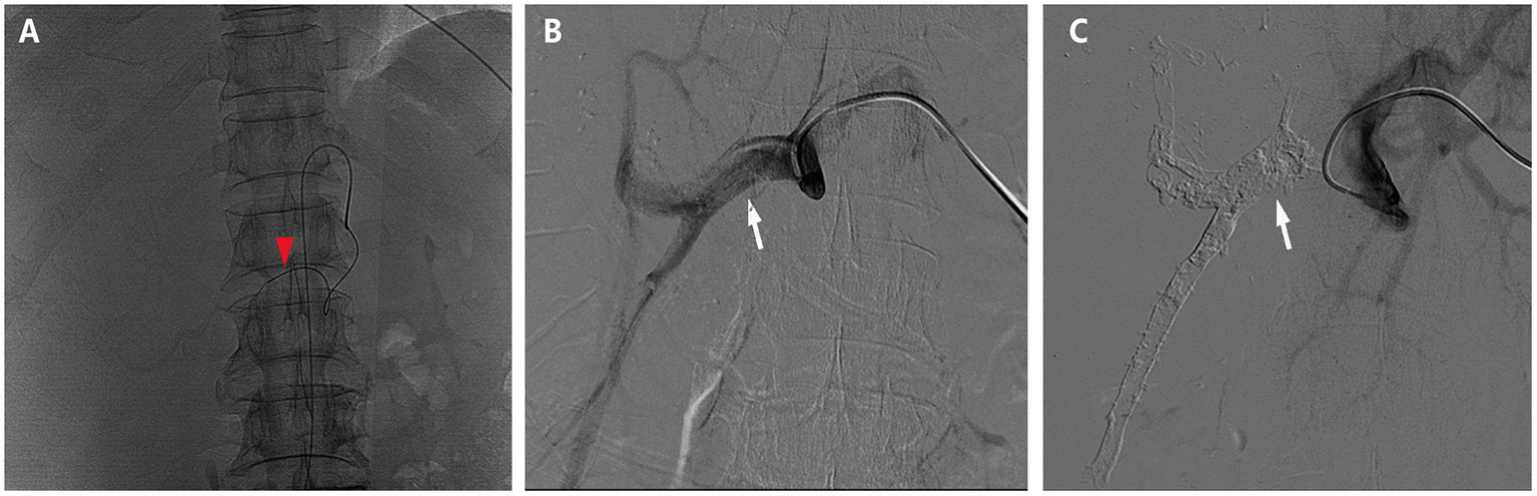
Hepatic artery infusion chemotherapy and portal vein embolization. **(A)** The tumor was mainly fed by both right and left hepatic arteries, so the infusion microcatheter was selectively placed into the common hepatic artery (the red arrow head). **(B)** The right branch of the portal vein before embolization (the white arrow). **(C)** The right branch of the portal vein after embolization (the white arrow).

**Figure 3:**
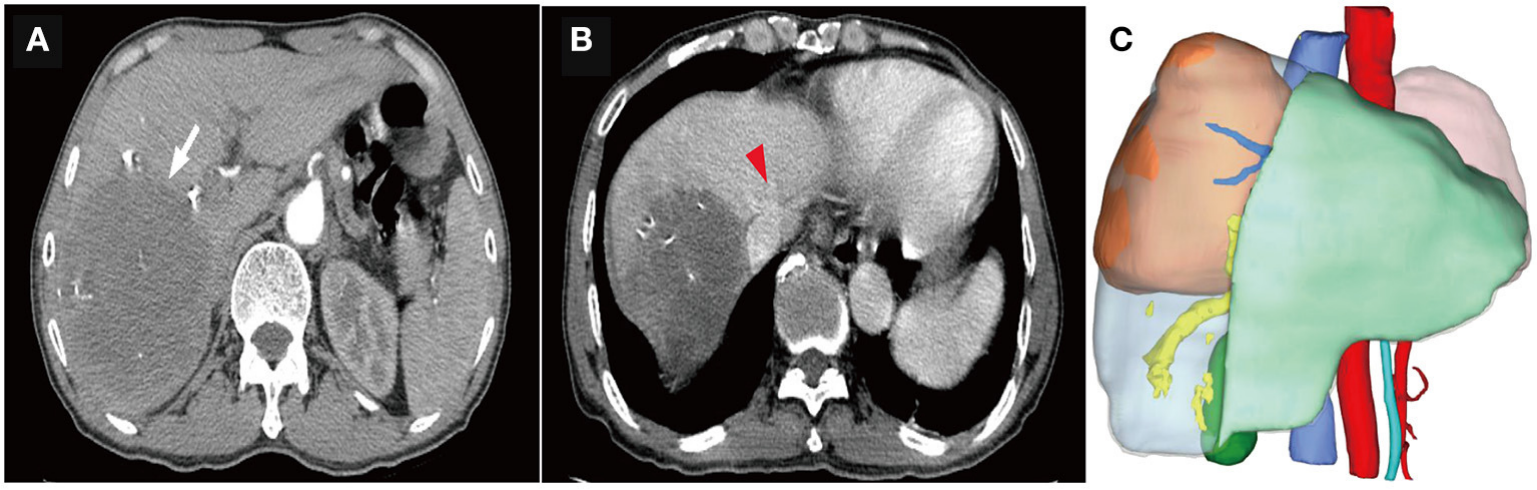
Computed tomography (CT) scans and three-dimensional reconstruction after portal vein embolization (PVE) and multimodality therapy. **(A)** The tumor remarkably regressed (the white arrow), with complete disappearance of intratumoral enhancement on the artery phase. **(B)** The distance between the tumor and the middle hepatic vein (the red arrow head) was significantly extended. **(C)** The ratio of future liver remnant (the light green area) to the standard liver volume (SLV) was 67.74%, which was well-tolerated for hepatectomy.

**Figure 4:**
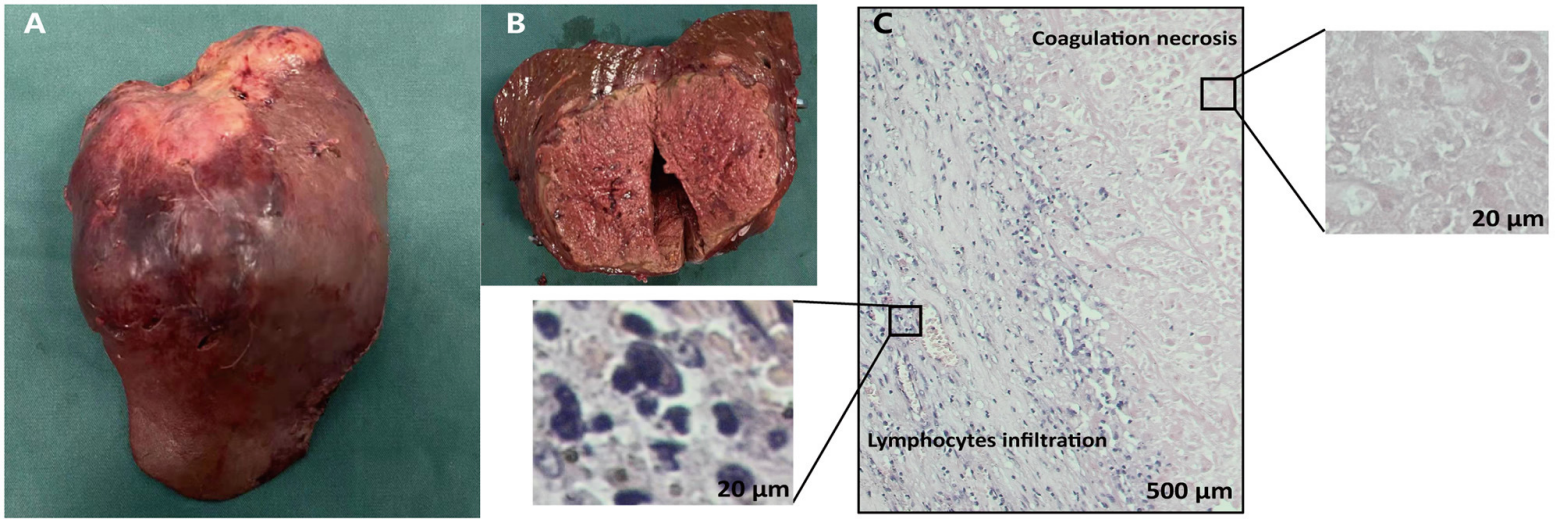
Gross and histopathological findings of the resected specimen. The gross **(A)** and cut **(B)** surface of the tumor showed the solid tumor with obvious hemorrhage and necrosis. **(C)** Microscopic finding of the liver mass showed abundant infiltration of lymphocytes and complete tumoral necrosis without any viable alive tumor cells.

**Figure 5:**
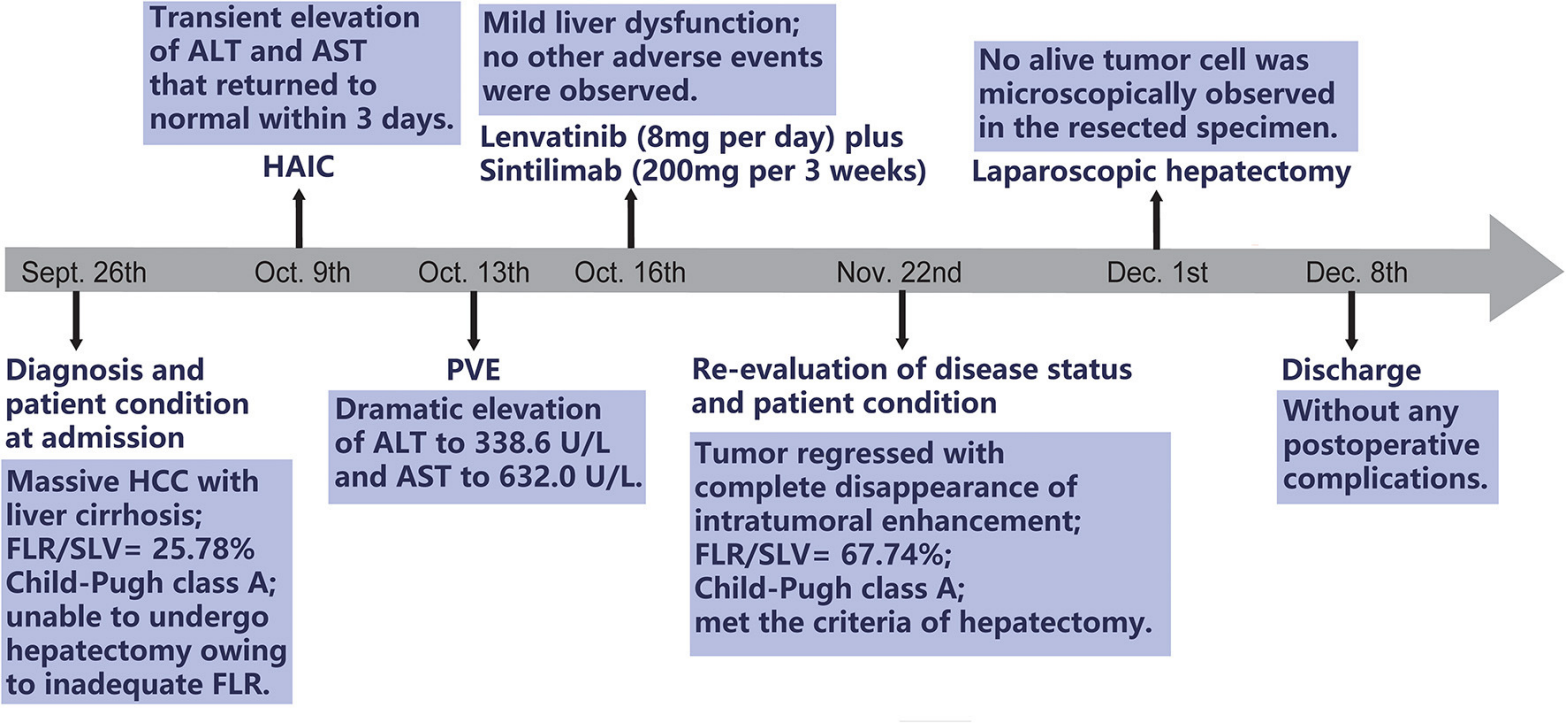
The timeline of clinical events. HCC, hepatocellular carcinoma; FLR, future liver remnant; SLV, standard liver volume; HAIC, hepatic artery infusion chemotherapy; ALT, alanine transaminase; AST, aspartate aminotransferase; PVE, portal vein embolization.

HAIC with the mFOLFOX regimen (oxaliplatin, 85 mg/m^2^ intra-arterial infusion; leucovorin, 400 mg/m^2^ intra-arterial infusion; and fluorouracil, 400 mg/m^2^ bolus infusion and 2,400 mg/m^2^ continuous infusion) was performed initially. The infusion microcatheter was selectively placed into the common hepatic artery, which was the main feeding artery to the tumor (Figure 2A). After 3 days of HAIC, biological glue was utilized to embolize the right branch of the portal vein (Figures 2B,C), resulting in a brief increase in alanine transaminase (ALT) to 338.6 U/L, aspartate aminotransferase (AST) to 632. U/L, and total bilirubin (TBIL) to 32.4 umol/L. Liver function normalized with medication within 3 days, and Lenvatinib (8 mg per day) plus Sintilimab (200 mg per 3 weeks) were administered subsequently, causing no significant adverse events. After 5 weeks of multimodality therapy, follow-up CT scans showed remarkable regression of the tumor with complete disappearance of intratumoral enhancement on the artery phase (Figure 3A) and increased distance between the tumor and the middle hepatic vein (Figure 3B). The FLR/SLV reached 67.74% (Figure 3C), and the Child-Pugh score was 5, which met the requirements of the surgery. One week later, laparoscopic right hemi-hepatectomy was successfully performed with a >1-cm surgical margin. The gross of the resected specimen showed obvious hemorrhage and necrosis of the tumor (Figures 4A,B). Postoperative pathology revealed an encapsulated tumor with massive hyalinization, necrosis, lymphocyte, and granulocyte infiltration, and no viable tumor cells (Figure 4C). The patient was discharged at the 7th day after the operation without any complications. He was advised to stop drinking and follow up regularly. At the 3-month follow-up, no significant evidence of recurrence on imaging or laboratory tests was observed. The timeline of clinical events is shown in Figure 5.

## Discussion

Surgery is considered the most essential radical treatment for solitary massive (≥10 cm) HCCs. However, owing both to the aggressiveness of HCCs and poor hepatic function reserve in many patients with underlying chronic liver disease, more than 60% of patients with HCC are ineligible for surgical intervention at initial diagnosis (10). In the present case, as the right-lobe tumor clung to the middle hepatic vein, extended right hemi-hepatectomy was required to ensure a sufficient surgical margin, which may result in inadequate FLR. For patients with inadequate FLR, conversion therapy is an appropriate approach to create the opportunity for surgical intervention. The conversion treatment should take into account both the increase in functional FLR and local control of the tumor.

Augmentation of FLR by PVE or ALPPS is a conventional available approach to reduce the risk of postoperative morbidity and mortality in potentially resectable HCCs (11). ALPPS can induce liver hypertrophy within 2 weeks, while PVE generally takes 4–6 weeks (5). Compared to PVE, ALPPS shows a higher conversion hepatectomy rate at a cost of higher postoperative morbidity and mortality (5), which is not feasible for the present case (age > 60 years) (12). However, for patients who received PVE only, the reported subsequent surgery rate ranged from only 60–80% (7). A retrospective study reported that combining PVE with TACE resulted in a 96% surgical rate (13). However, the efficacy of TACE on large (≥ 10 cm) HCC is limited, owing to multiple intrahepatic and/or extrahepatic collateral arterial supplies and arteriovenous fistulas (14). Some guidelines predominantly recommend trans-arterial radioembolization (TARE) to treat initially unresectable HCCs, with a conversion surgery rate of about 20.8–28.6% (15, 16). For patients undergoing TARE, the surgical resection is proposed to be considered at least 3 months later (15). In the present case, the patient wanted to complete surgery as soon as possible and, accordingly, did not consider TARE. Moreover, in China, radioactive microspheres are not currently reimbursed by the national health insurance system. Thus, high cost restricted the patient's choice of TARE (17). Recently, a prospective, non-randomized clinical trial based on Chinese populations has demonstrated a significant superiority of HAIC with the mFOLFOX regimen in terms of tumor response and a survival benefit in massive HCCs (8). HAIC is now widely used to treat initially unresectable HCCs in East Asia (18). The safety of PVE combined with HAIC in HCCs is rarely reported. In the present case, the level of ALT and AST briefly increased after the application of PVE and HAIC and decreased quickly within 3 days. The adverse effects on liver function were manageable.

Currently, HAIC-based comprehensive therapy, comprising TKI and PD-1 inhibitors, has shown encouraging preliminary results (19). A retrospective study revealed that a combination of HAIC and Lenvatinib plus PD-1 inhibitor reached an overall response rate of 40% and a disease control rate of 77.6% in advanced HCCs, mostly in BCLC stage C (20). Based on the retrospective investigation above, a Phase II, single-arm trial (NCT04044313) of Lenvatinib plus Toripalimab and HAIC as a first-line treatment for advanced hepatocellular carcinoma was carried out (21). Thirty-six patients were enrolled, with a median tumor size of 11.2 cm; 86.1% of the patients had portal vein invasion, and 27.8% had extrahepatic metastasis, indicating an extremely high tumor burden. The overall response rate was 66.7% according to the modified Response Evaluation Criteria in Solid Tumors (mRECIST), including eight patients eligible for surgical resection. A triple-combination regimen showed great conversion potential in advanced HCCs. To the best of our knowledge, the present case firstly reported the successful application of PVE plus triple combination regimen in massive, potentially resectable HCC. Notably, the patient received radiologic complete response after the PVE and multimodality therapy with mild and manageable adverse events.

In the present case, laparoscopic right hemi-hepatectomy was performed as the patient met the criteria for major hepatectomy. The major technical challenges during the operation are to ensure a sufficient surgical margin and to avoid the iatrogenic hematogenous spread of tumor cells. We used intraoperative ultrasound to guide the resection margin, and an anterior approach was conducted to avoid compression of the tumor. Postoperative morphological and histological examination showed that the resected specimen was intact and unbroken by a pathologically negative wide margin (>1 cm). Surprisingly, no viable alive tumor cells were observed, which may contribute to the long-term survival of the patient (22).

The present case provided a reference for clinicians in terms of inducing liver hypertrophy and simultaneous tumor control in treating potentially resectable HCCs. During liver hypertrophy, a combination of HAIC and Lenvatinib plus Sintilimab showed significant efficacy in depressing the tumor. The elderly patient showed good toleration of PVE and multimodality therapy, with manageable adverse events. Finally, the tumor was laparoscopically removed, with pCR. Follow-up will be continued to evaluate the long-term prognosis.

## Conclusion

This case demonstrates for the first time that combined PVE with multimodality therapy appears to be safe and effective for massive, potentially resectable HCC and can produce deep pathological remission in a primary tumor. More studies are needed to confirm the safety, feasibility, and efficacy of the new approach.

## Data Availability Statement

The original contributions presented in the study are included in the article/supplementary material, further inquiries can be directed to the corresponding authors.

## Ethics Statement

The studies involving human participants were reviewed and approved by Zhanjiang Central Hospital Ethics Committee. The patients/participants provided their written informed consent to participate in this study. Written informed consent was obtained from the individual(s) for the publication of any potentially identifiable images or data included in this article.

## Author Contributions

TH and CQ performed the surgery. DC performed the PVE and HAIC. QL designed the study and wrote the original draft. KL revised the manuscript. XF, QC, and WL collected and arranged imaging and pathological data. All authors contributed to the article and approved the submitted version.

## Conflict of Interest

The authors declare that the research was conducted in the absence of any commercial or financial relationships that could be construed as a potential conflict of interest.

## Publisher's Note

All claims expressed in this article are solely those of the authors and do not necessarily represent those of their affiliated organizations, or those of the publisher, the editors and the reviewers. Any product that may be evaluated in this article, or claim that may be made by its manufacturer, is not guaranteed or endorsed by the publisher.
